# New role for nuclear hormone receptors and coactivators in regulation of BRCA1-mediated DNA repair in breast cancer cell lines

**DOI:** 10.1186/bcr1362

**Published:** 2005-12-09

**Authors:** David L Crowe, Matt K Lee

**Affiliations:** 1 Center for Craniofacial Molecular Biology, University of Southern California, 2250 Alcazar Street, Los Angeles, CA 90033, USA

## Abstract

**Introduction:**

The breast cancer susceptibility gene *BRCA1 *is involved in the repair of double-strand breaks induced by ionizing radiation and chemotherapy drugs. BRCA1 interacts with coactivators such as p300 and CREB-binding protein (CBP) to activate target gene transcription. Estrogen and retinoic acid receptors (ER and RAR) also require coactivator proteins for their ligand-dependent functions. Few studies have suggested a role for nuclear hormone receptors in DNA repair.

**Methods:**

DNA damage and repair activity were quantified with the use of single-cell gel electrophoresis and plasmid end-joining assays. Cell cycle progression and apoptosis were determined by bromodeoxyuridine and TdT-mediated dUTP nick end labelling assays. Stable transfection was accomplished with the lipofection procedure. Protein interaction and expression were determined by immunoprecipitation and western blotting.

**Results:**

17β-Estradiol (E2) and all-*trans *retinoic acid (RA) had opposing effects on DNA damage and breast cancer cell survival after double-strand break damage. Treatment with E2, but not with RA, resulted in complex formation between ERα, CBP, and BRCA1 in ER-positive cell lines. Mutant BRCA1 reduced the expression and activity of DNA damage repair proteins but did not block nuclear hormone-dependent effects. Mutant BRCA1 failed to form complexes with ERα and CBP, which correlated with its ability to exert E2-independent effects on DNA repair. Mutant BRCA1 inhibited cell cycle progression and produced increased survival in cells with double-strand breaks. Ectopic ERα expression reproduced the E2-mediated effects on DNA damage, repair, and survival.

**Conclusion:**

The present study proposes a new mechanism by which ER and RAR regulate BRCA1-mediated DNA repair by means of CBP.

## Introduction

Breast cancer is one of the leading causes of death in women. Surgical removal of the tumor followed by radiation is the therapeutic mainstay for early disease [1]. Inactivating mutations in the tumor suppressor *BRCA1 *(breast cancer susceptibility gene 1) are associated with significantly increased risk of developing breast cancer [2]. The BRCA1 gene product contains a RING zinc-finger motif at the amino terminus and two BRCT (BRCA1 carboxyl-terminal) repeats [3]. The BRCT repeat is found in a range of proteins involved in DNA repair [4,5]. BRCA1 has been shown to regulate the DNA damage response [6-9]. BRCA1 is involved in repair of double-strand breaks induced by ionizing radiation and some chemotherapy drugs [10]. Double-strand breaks induce chromosomal abnormalities such as aneuploidy, deletions, and translocations, which are associated with cancer. Several chemotherapeutic agents used in the treatment of breast cancer produce their cytotoxic effects by creating DNA damage [11].

To repair double-strand breaks, mammalian cells use homologous recombination and end joining [12]. End joining is preferred in the G1 phase of the cell cycle when a second copy of the DNA strand is unavailable. Cells that have experienced double-strand breaks halt division and recruit repair factors such as Rad51, Mre11, and Nbs1 to damaged sites in DNA [13,14]. Mutations in double-strand break repair proteins give rise to human diseases that manifest as cancer predisposition, sensitivity to ionizing radiation, and chromosomal instability [15]. Mice containing null mutations in several of these factors exhibit chromosomal abnormalities and embryonic lethality [16].

Among the most important nuclear hormone receptors expressed by breast cancer cells are those for estrogen and retinoic acid [17]. Estrogens such as 17β-estradiol (E2) have been shown to markedly enhance the proliferation of mammary gland epithelium [18]. In contrast, several natural and synthetic retinoids have been shown to inhibit the proliferation of these cells and have been used as chemotherapy drugs in the treatment of breast cancer [19]. Estrogen receptors (ER) and retinoic acid receptors (RAR) are members of a family of ligand-dependent transcription factors that include steroid, thyroid, and vitamin D receptors [20]. Both ER and RAR have functional domains for DNA binding, ligand binding, dimerization, and transcriptional activation. Nuclear receptors and BRCA1 require coactivator proteins such as p300 and its close relative CREB-binding protein (CBP) to activate target gene transcription [21]. CBP/p300 interacts with ER and RAR in their ligand-bound conformation to induce gene expression [22]. CBP/p300 has histone acetyltransferase activity, permitting histone disassembly and transcriptional activation [23]. CBP/p300 has also been shown to interact with and enhance the function of BRCA1 [24].

Although the effects of E2 and all-*trans *retinoic acid (RA) on the proliferation of human breast cancer cells have been known for many years, only recently have gene expression profiling studies suggested a role for these hormones in DNA repair [25,26]. The present study proposes a new mechanism by which ER and RAR regulate BRCA1-mediated DNA repair via CBP.

## Materials and methods

### Cell culture

The human breast cancer cell lines used in this study (MCF7, T47D, MDA-MB-231, and MDA-MB-468) were purchased from the American Type Culture Collection and cultured in Dulbecco's modified Eagle's medium without phenol red (Invitrogen), 10% charcoal-resin-treated fetal bovine serum (Hyclone), and 40 μg/ml gentamicin (Invitrogen) in a humidified atmosphere of 5% CO_2 _at 37°C. Cultures were treated with 100 nM estradiol (Sigma), RA (Sigma), or vehicle for 8 hours before the addition of 30 μg/ml etoposide (Sigma) for 16 hours or a single 3 Gy dose of ionizing radiation (^60^Co source; USC/Norris Comprehensive Cancer Center) to induce DNA double-strand breaks. Treatment with 10 μg/ml cisplatin (which induces bulky adduct formation; Sigma) was used to control for type of DNA damage. To determine whether survival signals in this model system were mediated by protein kinases or second messengers, cultures were treated individually with 10 μM PD98059 (MEK inhibitor; Alexis Biochemicals), 5 μM SB203580 (p38 inhibitor; Alexis Biochemicals), 5 μM SP600125 (Jnk inhibitor; Calbiochem), 10 μM SH5 (Akt inhibitor, Alexis Biochemicals), 1 μM Go6976 (PKC inhibitor; Alexis Biochemicals), 10 μM LY294002 (phosphoinositide 3-kinase inhibitor; Alexis Biochemicals), or 1 μM U73122 (phospholipase Cγ inhibitor; Alexis Biochemicals) for 8 hours before addition of other compounds.

### Programmed cell death analysis

Cells were fixed in 4% paraformaldehyde (pH 7.4), and permeabilized with 0.1% Triton X-100 (Sigma) and 0.1% sodium citrate for 2 minutes on ice. A mouse IgM anti-human Fas antibody (Molecular Biology Laboratories, Woburn, MA, USA) that induces apoptosis in sensitive cell lines was used as the positive control (data not shown). An isotype-matched control antibody was used as the negative control. After being washed with PBS, cells were incubated with fluorescein-dUTP and terminal deoxynucleotidyl transferase for 60 minutes at 37°C in accordance with the manufacturer's recommendations (Roche Molecular Biochemicals). After being washed three times in PBS, apoptotic cells were revealed by fluorescence microscopy.

### DNA damage analysis

DNA damage was quantified by single-cell gel electrophoresis [27,28]. Drug-treated cells were mixed with 0.5% low-melting-point agarose and added to microscope slides coated with 1.5% agarose. Cells were denatured with alkali (pH 13), subjected to electrophoresis at 0.86 V/cm for 25 minutes, and stained with ethidium bromide. The tail moment (DNA migration × tail intensity) of 50 randomly selected cells was analyzed from each slide by using Komet imaging software (Kinetic Imaging).

### End joining assay

The well-characterized plasmid end-joining assay was used to evaluate nonhomologous end joining in breast cancer cell lines [29-31]. Reactions were performed in end-joining buffer containing 250 ng of dephosphorylated pBluescript II SK^- ^cut with *Eco*RI and *Xho*I restriction enzymes and 20 μg of T47D (ER^+^) or MDA-MB-468 (ER^-^) cellular extract in a final 20 μl volume at 25°C for 1 hour. The reactions were terminated and 20% of each sample was transformed into *E. coli *strain DH5α to quantify end-joining activity.

### Western blotting

Total cellular protein (75 μg) from breast cancer cell lines was separated by SDS-PAGE on 10% resolving gels under denaturing and reducing conditions. Separated proteins were electroblotted to poly(vinylidene difluoride) membranes in accordance with the manufacturer's recommendations (Roche Molecular Biochemicals). Blots were incubated with antibodies against human double-strand break repair or cell cycle regulatory proteins for 16 hours at 4°C. After being washed in Tris-buffered saline (pH 7.4) containing 0.1% Tween 20 (TBST), blots were incubated for 30 minutes at 20°C with anti-IgG secondary antibody conjugated to horseradish peroxidase. After extensive washing in TBST, bands were revealed by the enhanced chemiluminescence method (Roche Molecular Biochemicals).

### Immunoprecipitation

Cultures were lysed in 50 mM HEPES (pH 7.5), 150 mM NaCl, 1 mM EDTA, 2.5 mM EGTA, 1 mM dithiothreitol, 1% Nonidet P40, 10% glycerol, 1 mM NaF, 0.1 mM sodium orthovanadate, and protease inhibitors for 30 minutes at 4°C. Lysates were centrifuged at 10,000 *g *for 10 minutes, and anti-CBP antibody (Santa Cruz Biotechnology) was incubated with the supernatants for 1 hour at 4°C. Preimmune IgG was used as a negative control antibody for immunoprecipitation. Antigen-antibody complexes were precipitated with anti-CBP antibody and protein A/G agarose beads for 1 hour at 4°C. Immunoprecipitated proteins were washed three times with 1 ml of lysis buffer. Samples were boiled in Laemmli buffer for 3 minutes, separated by SDS-PAGE, and blotted to poly(vinylidene difluoride) membranes. Blots were incubated with anti-ERα, anti-RARα, and anti-BRCA1 antibodies followed by anti-CBP antibody to ensure equal amounts of immunoprecipitated protein in each lane.

### Transfections

T47D and MDA-MB-468 cells were stably transfected with a BRCA1 mutant construct lacking the carboxyl-terminal 276 amino acid residues containing the BRCT repeat region (BRCA1 construct kindly provided by Dr Kenneth Cowan) or G418 resistance plasmid with the use of Lipofectamine reagent in accordance with the manufacturer's recommendations (Invitrogen). MDA-MB-468 cells were separately transfected with an ERα expression vector (kindly provided by Dr Ronald Evans) or G418 resistance plasmid. Cells were selected in 400 μg/ml G418 for 14 days. Resistant clones were picked for expansion and characterization. Separate cultures were transiently transfected with 50 nM BRCA1 short interfering RNA (siRNA; Santa Cruz Biotechnology) or unrelated siRNA with the use of Lipofectamine, before being harvested for further analysis.

### Bromodeoxyuridine incorporation analysis

Cells were incubated with 10 μM bromodeoxyuridine (BrdU) for 1 hour. After being washed in PBS, cells were fixed in 70% ethanol, 50 mM glycine (pH 2) for 30 minutes at -20°C. After extensive washing in PBS, cells were incubated with mouse anti-BrdU primary antibody at 37°C for 30 minutes (Roche Molecular Biochemicals). After being washed in PBS, cells were incubated with anti-mouse IgG secondary antibody conjugated to fluorescein at 37°C for 30 minutes. After extensive washing in PBS, BrdU-positive cells were revealed by fluorescence microscopy. The number of positive cells was expressed as a percentage of total cells counted in 10 high-power fields.

### Statistical analysis

Parametric data were analyzed by *t *test and analysis of variance; *p *< 0.05 was considered statistically significant. All experiments were performed at least three times.

## Results

**Figure 1:**
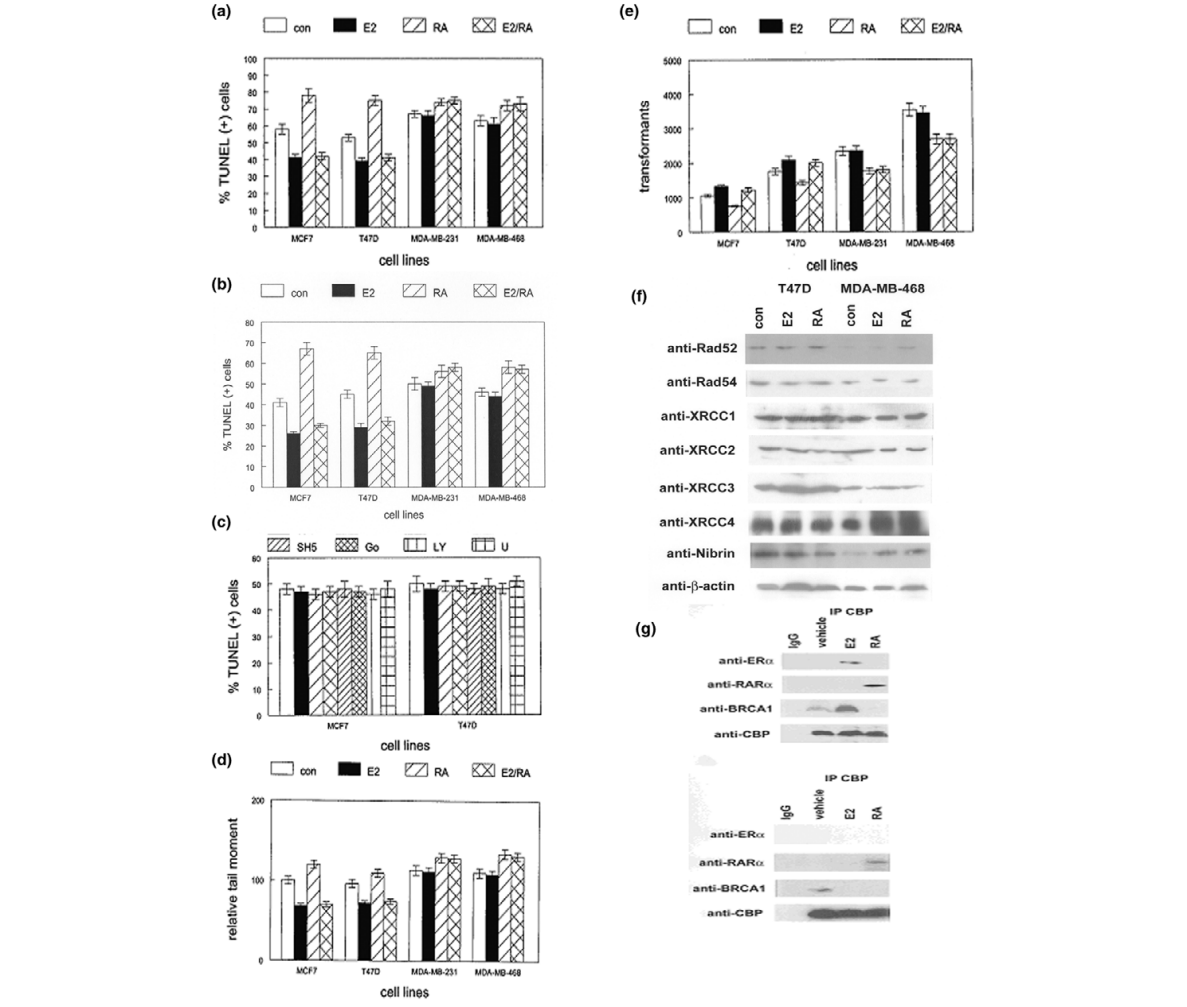
E2 inhibits and RA enhances DNA damage-mediated apoptosis in human breast cancer cell lines. **(a) **Estrogen receptor (ER)-positive human breast cancer cell lines (MCF7 and T47D) and ER-negative lines (MDA-MB-231 and MDA-MB-468) were treated with 17β-estradiol (E2) or all-*trans *retinoic acid (RA) alone or in combination (E2/RA) prior to etoposide induced DNA damage. Vehicle-treated cells were used as the negative control (con). Apoptotic cells were identified by TdT-mediated dUTP nick end labeling (TUNEL) assay. Error bars indicate SEM. **(b) **The human breast cancer cell lines identified above were treated with E2 or RA alone or in combination (E2/RA)before treatment with 3 Gy of ionizing radiation to induce double-strand DNA breaks. Vehicle-treated cells were used as the negative control (con). Apoptotic cells were identified by TUNEL assay. Error bars indicate SEM. **(c) **The pro-survival effects of E2 were not mediated by MAPK, PKC, phosphoinositide 3-kinase, phospholipase Cγ, or Akt/PKB signaling in human breast cancer cell lines. ER-positive breast cancer cell lines MCF7 and T47D were pretreated with PD98059 (PD), SP600125, (SP), SB203580 (SB), SH5, (Akt/PKB inhibitor), Go6976 (Go), LY294002 (LY), or U73122 (U) as indicated in Materials and Methods. Vehicle-treated cells were used as the negative control (con) for the TUNEL assay. **(d) **E2 inhibits and RA increases the extent of DNA damage in etoposide-treated human breast cancer cell lines. The indicated ER-positive and ER-negative cell lines were treated with E2 or RA alone or in combination (E2/RA), as described above, before exposure to etoposide. Vehicle-treated cells were used as the negative control (con). Relative DNA damage was quantified as described in Materials and Methods. **(e) **E2 enhances and RA inhibits DNA double-strand break repair in human breast cancer cell lines. The indicated cell lines were treated with E2 or RA alone or in combination (E2/RA) as described above. Vehicle-treated cells were used as the negative control (con). The plasmid end-joining assay was used to quantify DNA repair activity. **(f) **E2 or RA treatment does not affect double-strand break repair protein expression in human breast cancer cell lines. The indicated lines were treated with E2 or RA as described above. Vehicle-treated cells were used as the negative control (con). Protein extracts from treated cells were subjected to western blotting with the anti-human antibodies indicated at the left. **(g) **Complex formation of BRCA1 with CBP and ERα in E2-treated but not RA-treated T47D cells (upper panel). Vehicle-treated cells were used as the negative control. The CBP coactivator protein was immunoprecipitated from treated cultures with anti-CBP antibody (IP CBP). Preimmune IgG was used as the negative control for immunoprecipitation. Immunoprecipitates were subjected to western blotting to detect interaction with ERα, retinoic acid receptor α (RARα), and BRCA1 by using the antibodies indicated at the left. Blots were stripped and incubated with anti-CBP antibody to detect relative amounts of immunoprecipitated protein in each lane. These experiments were performed three times with similar results; representative blots are shown.

We treated four human breast cancer cell lines with 100 nM E2 or RA, followed by etoposide to induce double-strand DNA breaks. As shown in Fig. 1a, treatment with etoposide resulted in 60 to 70% TdT-mediated dUTP nick end labelling (TUNEL)-positive cells within 16 hours. Pretreatment with E2 resulted in increased survival of ER-positive MCF7 and T47D breast cancer cell lines (40% TUNEL-positive cells; *p *< 0.005) compared with vehicle-treated control cultures. No effect of E2 was observed in ER-negative MDA-MB-231 and MDA-MB-468 cell lines. In contrast, treatment with RA increased the number of apoptotic cells to 80% in all cell lines (*p *< 0.001). In cultures simultaneously treated with E2 and RA, the pro-survival effect of E2 was still observed in ER-positive cells but not in ER-negative lines. Similar effects of these ligands were observed when ionizing radiation was used to induce double-strand breaks (Fig. 1b). However, no effects of E2 or RA were observed in cisplatin-treated cultures, indicating that the effects of these ligands were specific for survival after double-strand breaks but not adduct formation (data not shown). We concluded that E2 and RA had opposing effects on breast cancer cell survival after double-strand DNA break damage.

To determine whether the pro-survival effects of E2 were mediated by kinase signaling or by second messengers, we treated ER-positive MCF7 and T47D cells with selective inhibitors of these pathways before treatment with E2 and etoposide. As shown in Fig. 1c, treatment with MEK, JNK, p38, Akt, PKC, phosphoinositide 3-kinase, or phospholipase Cγ inhibitors had no effect on the pro-survival effect of E2 as determined by TUNEL assay. These results indicate that signaling pathways upstream of ER do not regulate the pro-survival effect of E2 in cells exposed to DNA double-strand break damage.

To determine whether the effects of E2 and RA on cell survival were correlated with the extent of double-strand break damage, we performed single-cell gel electrophoresis on human breast cancer cell lines treated with these ligands before etoposide. As shown in Fig. 1d, E2 decreased the extent of DNA damage by 40% in ER-positive cell lines (*p *< 0.03). No effect of E2 on DNA damage was observed in ER-negative cell lines. In contrast, RA increased relative DNA damage levels by 10 to 20% in all cell lines examined (*p *< 0.01). In cells treated simultaneously with E2 and RA, relative DNA damage levels decreased by an amount similar to that after treatment with E2 alone. These results indicate that the cell survival effects of E2 and RA on human breast cancer cell lines are correlated with relative DNA damage levels in cultures treated with these ligands followed by etoposide.

To determine whether effects of E2 and RA on DNA damage could result from changes in DNA repair activity, we analyzed plasmid end joining in ligand-treated human breast cancer cell lines. As shown in Fig. 1e, E2 increased the number of transformants in the end joining assay by 20% when extract from ER-positive cell lines was used (*p *< 0.006). No effect of E2 was observed with ER-negative cell extract. Treatment with RA inhibited plasmid end joining in all cell extracts by 30% (*p *< 0.002). In extracts from cells treated simultaneously with E2 and RA, the number of transformants increased by an amount similar to that after treatment with E2 alone with extract from ER-positive cells. These results indicate that the effects of E2 and RA on DNA damage were correlated with DNA repair activity in human breast cancer cell lines.

To determine whether the effects of E2 and RA on DNA repair activity were the result of changes in repair protein expression, we examined the expression of double-strand break repair gene products by western blotting. As shown in Fig. 1f, E2 and RA did not affect the expression of seven double-strand break repair proteins in ER-positive and ER-negative human breast cancer cell lines. These results indicated that the effects of E2 and RA on DNA repair activity were not the result of changes in repair protein expression. We therefore wondered whether ER and RAR coactivator proteins such as CBP might differentially associate with these receptors and regulators of DNA repair such as BRCA1 in human breast cancer cell lines. As shown in Fig. 1g, treatment with E2 induced complex formation between ERα, BRCA1, and CBP in ER-positive T47D cells (upper panel). This complex was not observed in ER-negative MDA-MB-468 cells treated with E2 (lower panel). Treatment with RA showed recruitment of CBP to RARα in both cell lines, but BRCA1 was not detected in these complexes. Low-level association of BRCA1 with CBP was observed in vehicle-treated cells, but neither ERα nor RARα was detected in these complexes. No protein interactions were observed when preimmune IgG was used in place of anti-CBP antibody in the immunoprecipitations. These results indicate that treatment with E2 results in complex formation between ERα, CBP, and BRCA1 in ER-positive breast cancer cell lines; treatment with RA recruits CBP but not BRCA1 to RARα in both ER-positive and ER-negative cell lines.

**Figure 2:**
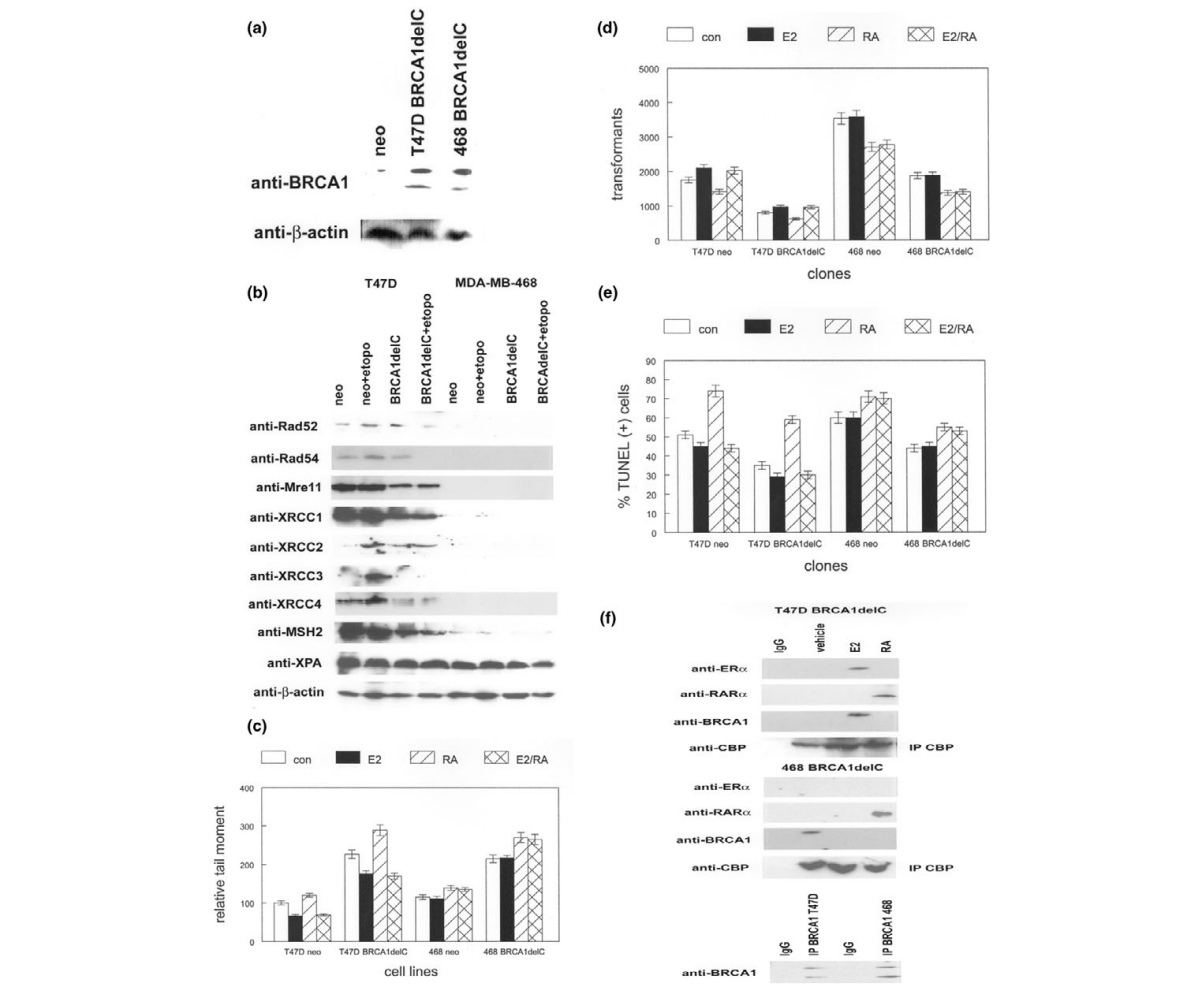
BRCA1 inhibition decreases DNA damage repair and cell survival in human breast cancer cell lines. **(a) **Expression of dominant-negative BRCA1 in human breast cancer cell lines. T47D and MDA-MB-468 cells were stably transfected with the BRCA1 carboxyl-terminal truncation mutant (BRCA1delC) or neomycin expression vector (neo). Protein extracts from these clones were subjected to western blotting with anti-BRCA1 antibody. Anti-β-actin antibody was used to determine relative amounts of protein in each lane. Representative blots are shown. **(b) **BRCA1 inhibition decreases the expression of double-strand break repair proteins in human breast cancer cell lines. T47D or MDA-MB-468 BRCA1delC or neomycin-resistant control (neo) clones were treated with etoposide (etopo) or vehicle prior to western blotting with antibodies indicated at the left. Blots were stripped and incubated with anti-β-actin antibody to determine relative amounts of protein in each lane. Representative blots are shown. **(c) **T47D and MDA-MB-468 BRCA1delC clones exhibit increased DNA damage when treated with etoposide. BRCA1delC or neomycin-resistant control clones (neo) were pretreated with E2RA alone or in combination (E2/RA) before exposure to etoposide. Vehicle-treated cultures were used as the negative control (con). Relative DNA damage was quantified as described in Materials and Methods. Error bars indicate SEM. **(d) **T47D and MDA-MB-468 BRCA1delC clones exhibit decreased DNA repair activity. BRCA1delC or neomycin-resistant control clones (neo) were pretreated with E2 or RA alone or in combination (E2/RA). Vehicle-treated cultures were used as the negative control (con). The plasmid end-joining assay was used to quantify DNA repair activity. Error bars indicate SEM. **(e) **Increased cell survival in etoposide-treated T47D and MDA-MB-468 BRCA1delC clones. Neomycin control clones were used as controls. Cultures were pretreated with E2 or RA alone or in combination (E2/RA). Vehicle-treated cultures were used as the negative control (con). Cell death was quantified by TdT-mediated dUTP nick end labelling assay. These experiments were repeated three times with similar results. Error bars indicate SEM. **(f) **No complex formation of BRCA1delC with CBP and estrogen receptor α (ERα) in T47D and MDA-MB-468 cells. Stable clones expressing BRCA1delC were treated with E2 or RA as described in Materials and Methods. Vehicle-treated cells were used as the negative control. The CBP coactivator protein was immunoprecipitated from treated cultures with anti-CBP antibody (IP CBP). Preimmune IgG was used as the negative control for immunoprecipitation. Immunoprecipitates were subjected to western blotting to detect interaction with ERα, retinoic acid receptor α (RARα), and BRCA1 by using the antibodies indicated at the left. Blots were stripped and incubated with anti-CBP antibody to detect relative amounts of immunoprecipitated protein in each lane. In contrast, both wild-type and mutant BRCA1 immunoprecipitated from both cell lines with anti-BRCA1 antibody (lower panel). These experiments were performed three times with similar results; representative blots are shown.

Given that recruitment of BRCA1 to the ERα/CBP complex was correlated with increased DNA repair and survival, which was not observed in RA-treated cells, we wished to determine the contribution of BRCA1 to these processes. To accomplish this task, we stably transfected T47D and MDA-MB-468 breast cancer cells with a carboxyl-terminal truncation mutant of BRCA1. This BRCA1 mutant lacked the BRCT repeat region believed to be involved in DNA repair [4,5]. Expression of the endogenous BRCA1 gene product and the mutant construct is shown by the western blot in Fig. 2a. To determine the effects of the BRCA1 mutant on the expression of double-strand break repair proteins, we treated stable T47D and MDA-MB-468 mutant and control clones with etoposide for 16 hours. As shown in Fig. 2b, treatment with etoposide induced the expression of Rad52, Rad54, XRCC2, XRCC3, and XRCC4 in T47D control clones. The mutant BRCA1 protein blocked the induction of all five of these genes by etoposide. In contrast, expression of the mismatch repair protein MSH2 and the nucleotide excision repair gene product XPA was unaffected by treatment with the mutant BRCA1 or etoposide. Similar effects of the BRCA1 mutant were observed with ER-positive MCF7 and ER-negative MDA-MB-231 cells (data not shown). These results indicate that mutant BRCA1 inhibits the expression of DNA damage repair proteins in human breast cancer cell lines.

To determine whether the mutant BRCA1 protein could block the protective effects of E2 on ER-positive breast cancer cell lines, we treated T47D stable clones with E2 or RA followed by etoposide. The ER-negative MDA-MB-468 clones served as controls in these experiments. As shown in Fig. 2c, E2 (*p *< 0.002) and RA (*p *< 0.008) reproduced the effects on relative DNA damage levels in T47D control clones first seen in untransfected cells. In contrast, relative DNA damage levels were twofold higher in T47D clones expressing the mutant BRCA1 protein (*p *< 0.0002). However, the mutant BRCA1 was unable to block either the protective effects of E2 or the deleterious effects of RA on relative DNA damage levels in these cells. The E2 effect again dominated in cultures treated simultaneously with E2 and RA. DNA damage was also greater in ER-negative MDA-MB-468 clones expressing mutant BRCA1 but was unresponsive to E2. Treatment with RA increased relative DNA damage levels by 20% in these clones. These results indicate that mutant BRCA1 expression was correlated with increased etoposide-mediated DNA damage in human breast cancer cell lines but did not block nuclear hormone-dependent effects.

To determine whether increased DNA damage as the result of mutant BRCA1 resulted from decreased repair activity, we used lysates from E2 and RA breast cancer clones in the end-joining assay. As shown in Fig. 2d, expression of the BRCA1 mutant decreased end joining by 60% with lysate from T47D clones (*p *< 0.04). The mutant BRCA1 gene product did not block the effects of E2 and RA on end joining in this assay. Expression of the mutant BRCA1 also decreased end joining in MDA-MB-468 clones by 50% (*p *< 0.04). Treatment of these clones with RA produced a 25% reduction in end joining in these assays (*p *< 0.02), but treatment with E2 had no effect in the ER-negative clones. These results indicated that expression of the BRCA1 mutant resulted in decreased DNA repair activity in ER-positive and ER-negative breast cancer clones.

We expected the decreased DNA repair activity observed in BRCA1 mutant clones to correlate with decreased survival in breast cancer cells exposed to etoposide. However, as shown in Fig. 2e, expression of the BRCA1 mutant resulted in increased survival of both T47D and MDA-MB-468 clones. Etoposide treatment produced only 35% TUNEL-positive cells in T47D clones expressing the BRCA1 mutant construct, compared with 50% in control cultures (*p *< 0.0005). Similarly, etoposide treatment resulted in 45% TUNEL-positive MDA-MB-468 mutant cells, compared with 60% of control clones (*p *< 0.03). The pro-survival effects of E2 and pro-apoptotic effects of RA were not blocked by the BRCA1 mutant in T47D clones. RA also had pro-apoptotic effects on MDA-MB-468 clones expressing the BRCA1 mutant, but E2 had no effect on the ER-negative line. These results indicate that despite decreased DNA repair as the result of mutant BRCA1, this construct also produced increased survival in breast cancer cells with DNA double-strand breaks.

We hypothesized that the failure of the mutant BRCA1 protein to affect E2-mediated DNA repair might have been due to decreased ability of the truncated tumor suppressor to interact with CBP. To test this hypothesis we immunoprecipitated CBP from E2-treated and RA-treated stable T47D and MDA-MB-468 clones expressing the truncated BRCA1 protein. As shown in Fig. 2f, the larger wild-type BRCA1 protein immunoprecipitated with CBP in both T47D and MDA-MB-468 clones. However, the mutant BRCA1 protein was not detected in these immunoprecipitates even though it was detected in these cells when anti-BRCA1 antibody was used in the immunoprecipitation. ERα formed complexes with wild-type BRCA1 and CBP in E2-treated T47D clones but not in MDA-MB-468 clones, a similar pattern to that observed in the parental breast cancer cell lines. RARα became associated with CBP but not with wild-type BRCA1 in RA-treated T47D and MDA-MB-468 clones. These results indicate that the truncated BRCA1 fails to form complexes with ERα and CBP, which correlates with its ability to exert E2 -independent effects on DNA damage repair.

**Figure 3:**
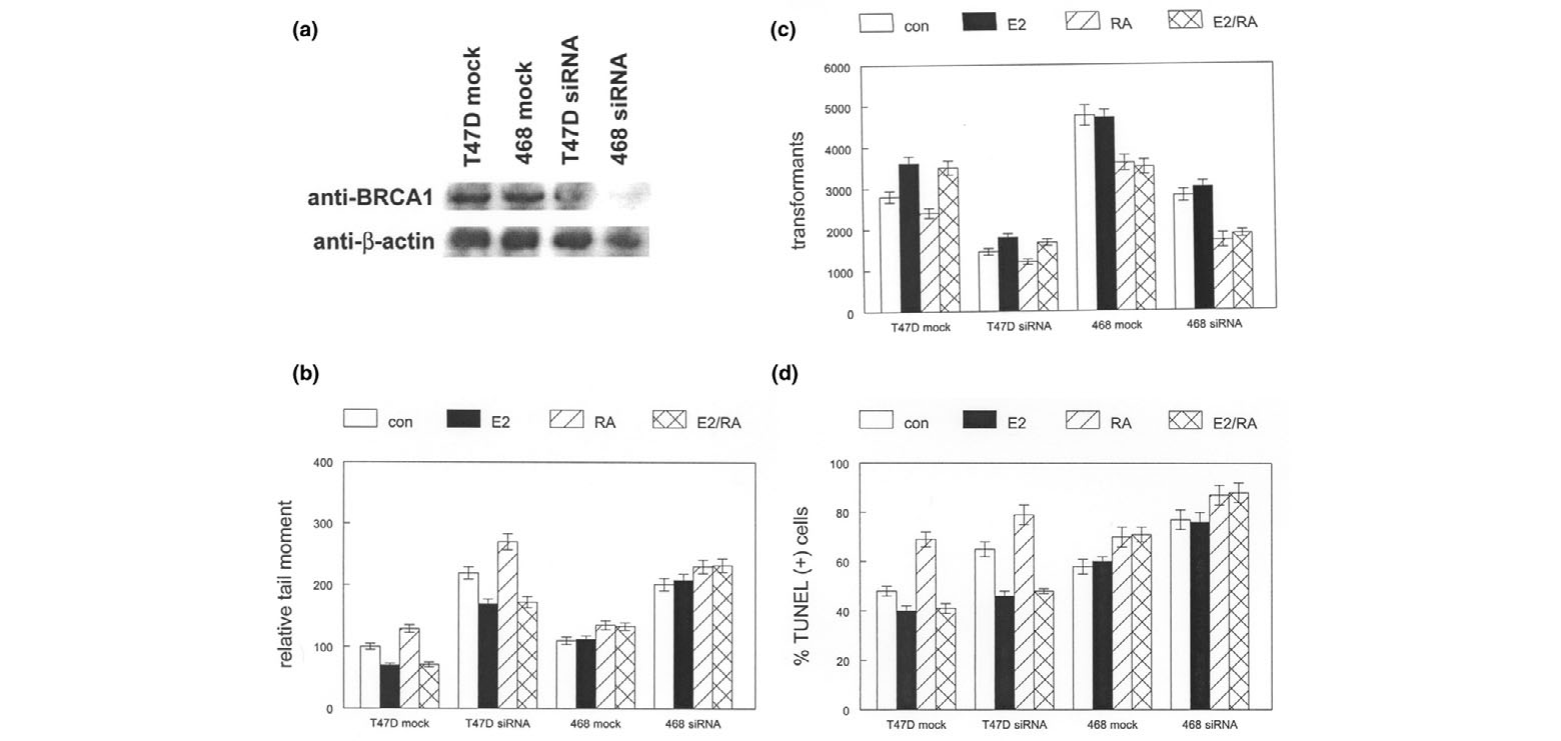
BRCA1 depletion decreases DNA damage repair and cell survival in human breast cancer cell lines. **(a) **Reduced BRCA1 expression in human breast cancer cell lines transfected with siRNA. T47D and MDA-MB-468 cells were transiently transfected with BRCA1 siRNA or an unrelated siRNA (mock). Protein extracts from these clones were subjected to western blotting with anti-BRCA1 antibody. Anti-β-actin antibody was used to determine relative amounts of protein in each lane. Representative blots are shown. **(b) **Decreased BRCA1 expression results in increased DNA damage when treated with etoposide. T47D and MDA-MB-468 lines transfected with BRCA1 or unrelated siRNA (mock) were pretreated with 17β-estradiol (E2) or all-*trans *retinoic acid (RA) alone or in combination (E2/RA) before exposure to etoposide. Vehicle-treated cultures were used as the negative control (con). Relative DNA damage was quantified as described in Materials and Methods. Error bars indicate SEM. **(c) **Decreased BRCA1 expression results in decreased DNA repair activity. T47D or MDA-MB-468 lines transfected with BRCA1 or unrelated siRNA (mock) were pretreated with E2 or RA alone or in combination (E2/RA). Vehicle-treated cultures were used as the negative control (con). The plasmid end-joining assay was used to quantify DNA repair activity. Error bars indicate SEM. **(d) **Decreased BRCA1 expression results in decreased cell survival in etoposide-treated T47D and MDA-MB-468 lines transfected with BRCA1 siRNA. Cultures transfected with unrelated siRNA (mock) were used as controls. Cultures were pretreated with E2 or RA alone or in combination (E2/RA). Vehicle-treated cultures were used as the negative control (con). Cell death was quantified by TdT-mediated dUTP nick end labelling assay. These experiments were repeated three times with similar results. Error bars indicate SEM.

To confirm that loss of function was responsible for the effects of the BRCA1 mutant, we transfected cultures of breast cancer cell lines with BRCA1 siRNA. As shown in Fig. 3a, siRNA transfection reduced BRCA1 protein expression by more than 90% in T47D and MDA-MB-468 cells. Decreased BRCA1 expression doubled the relative DNA damage in both cell lines (*p *< 0.0001) but did not block hormone-dependent effects (Fig. 3b). BRCA1 siRNA transfection inhibited DNA damage repair in both cell lines by 40 to 50% (*p *< 0.05) but did not block hormone-dependent effects (Fig. 3c). However, decreased BRCA1 expression resulted in increased cell death after exposure to etoposide (Fig. 3d; *p *< 0.05 for T47D, *p *< 0.01 for MDA-MB-468). These results indicate that BRCA1 loss of function produces increased DNA damage and cell death as a result of reduced repair capability.

**Figure 4:**
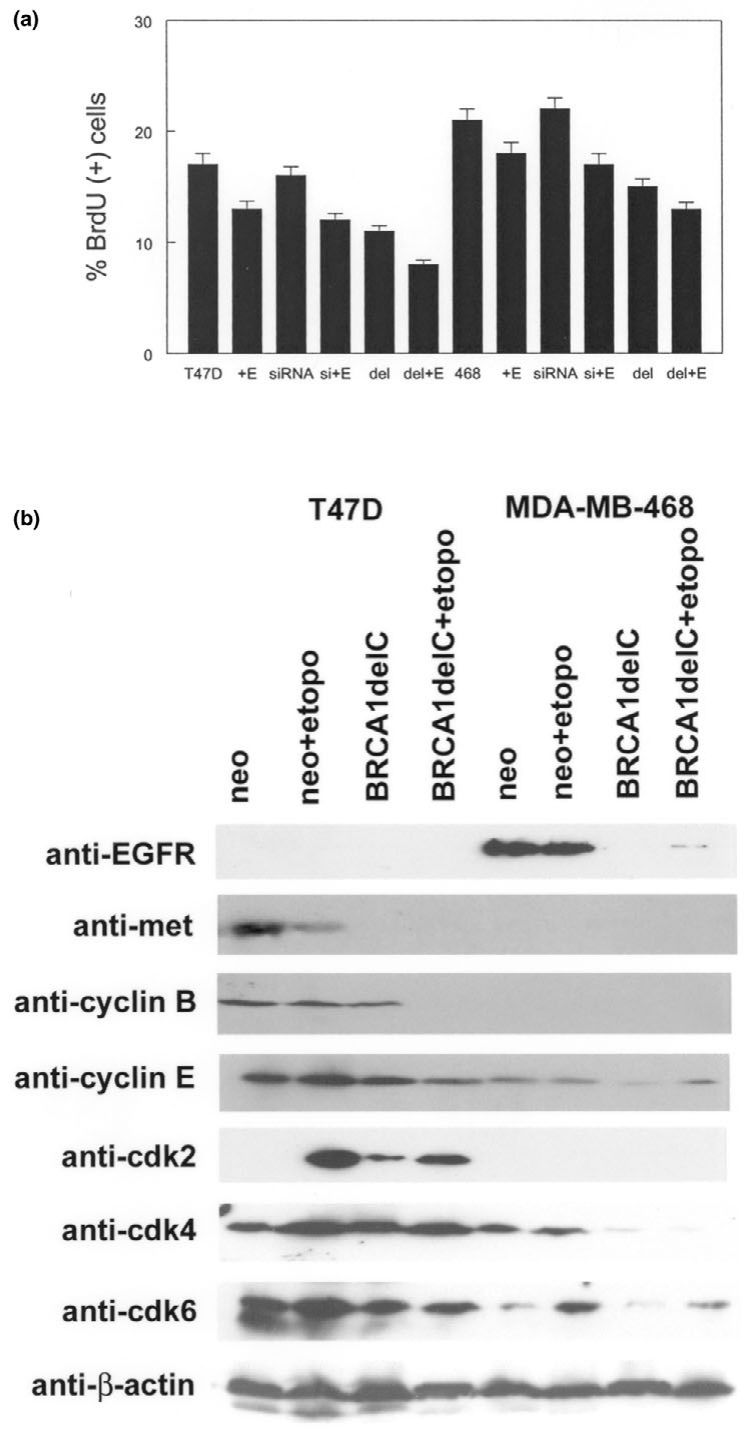
BRCA1delC, but not siRNA, inhibits cell cycle progression in human breast cancer cell lines. **(a) **Decreased bromodeoxyuridine (BrdU) incorporation in T47D and MDA-MB-468 BRCA1delC (del) clones. Neomycin-resistant clones were used as controls. Separate cultures were transiently transfected with BRCA1 siRNA. Clones were treated with etoposide (E) as described in Materials and Methods. These experiments were performed three times with similar results. Error bars indicate SEM. **(b) **T47D and MDA-MB-468 BRCA1delC and neomycin-resistant control (neo) clones were treated with etoposide (etopo) or vehicle prior to western blotting with the anti-cell-cycle-regulatory antibodies indicated at the left. Blots were stripped and incubated with anti-β-actin antibody to determine the relative amounts of protein in each lane. Representative blots are shown.

Given that DNA damage agents target dividing cells, we hypothesized that cell cycle inhibition due to the mutant BRCA1 could result in greater resistance to etoposide. BrdU incorporation analysis demonstrated that the mutant BRCA1 transgene (but not siRNA treatment) inhibited S-phase progression in both T47D (17% versus 10% positive cells; *p *< 0.03) and MDA-MB-468 (20% versus 16%; *p *< 0.02) lines (Fig. 4a). The effect of the BRCA1 mutant was greater than that of treatment of control clones with etoposide. Treatment of BRCA1 clones with etoposide further reduced BrdU incorporation (17% versus 7% positive cells in T47D and 20% versus 13% in MDA-MB-468 lines). We also examined the expression of cell cycle regulatory proteins in both lines (Fig. 4b). Expression of the mutant BRCA1 reduced epidermal growth factor receptor levels below the limit of detection for western blotting in MDA-MB-468 clones. Similarly, expression of the growth factor receptor c-Met was completely inhibited in T47D clones expressing mutant BRCA1. Expression of the G2-phase protein cyclin B was reduced to undetectable levels in etoposide-treated T47D clones expressing the mutant BRCA1 construct. Expression of the G1-phase protein cyclin E was inhibited twofold in T47D clones expressing the mutant BRCA1. Treatment with etoposide induced cyclin-dependent kinase (Cdk)2 levels in these clones, which was inhibited fivefold by the mutant BRCA1. This construct also reduced expression of the G1 kinases Cdk4 and Cdk6 to nearly undetectable levels in MDA-MB-468 clones. These results indicate that the mutant BRCA1 construct inhibited cell cycle progression, which correlated with increased resistance to etoposide.

**Figure 5:**
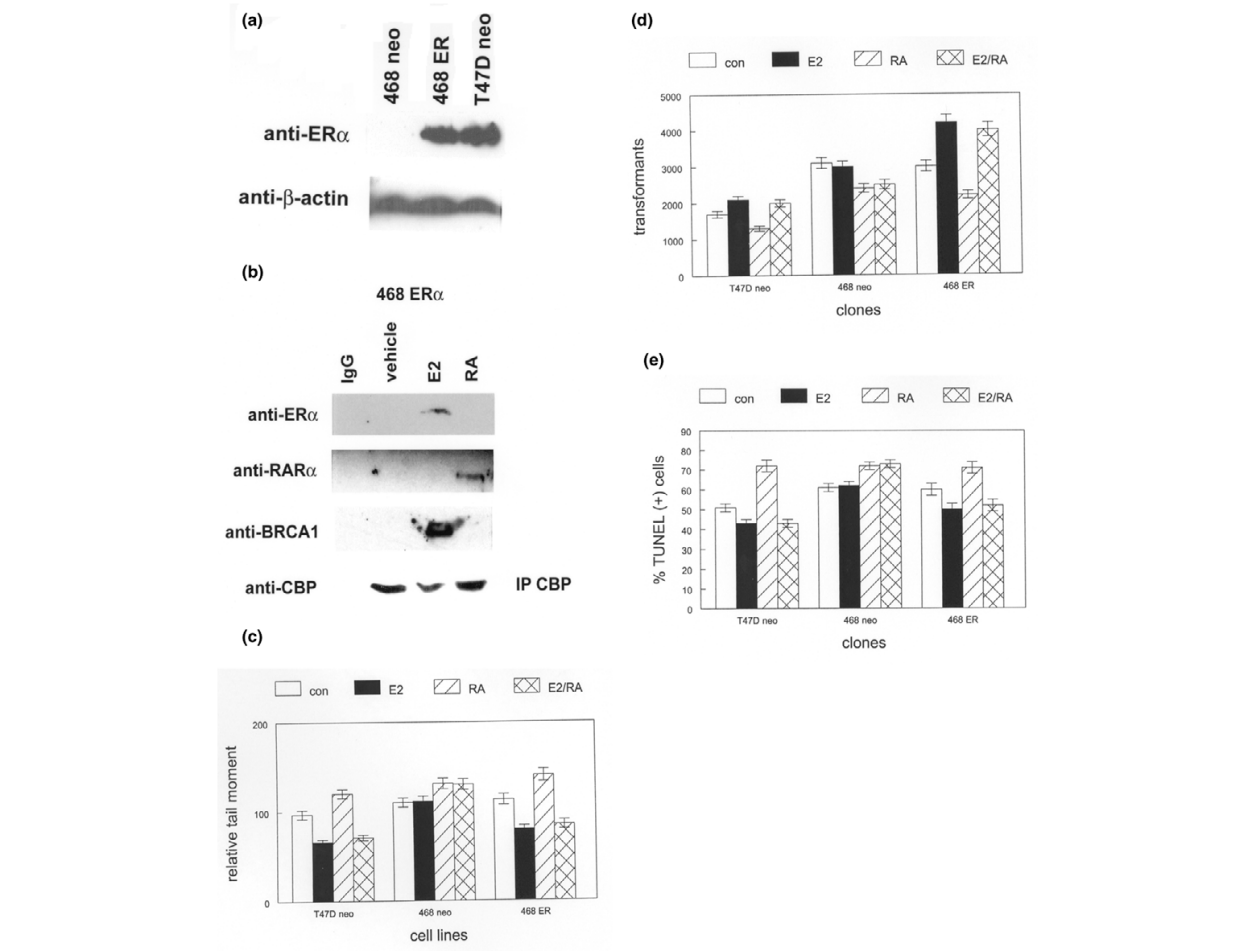
E2-dependent effects on DNA damage and cell survival are mediated by the expression of ERα. **(a) **ER-negative MDA-MB-468 cells were stably transfected with estrogen receptor α (ERα) expression vector (ER) or neomycin resistance plasmid (neo). ER-positive T47D neomycin-resistant control clones (neo) were used for comparison of ERα expression. Blots were stripped and incubated with anti-β-actin antibody to determine relative amounts of protein in each lane. **(b) **Complex formation of BRCA1 with CBP and ERα in MDA-MB-468 ERα stable clones treated with E2 but not with RA. Vehicle-treated cells were used as the negative control. The CBP coactivator protein was immunoprecipitated from treated cultures with anti-CBP antibody (IP CBP). Preimmune IgG was used as the negative control for immunoprecipitation. Immunoprecipitates were subjected to western blotting to detect interaction with ERα, retinoic acid receptor α (RARα), and BRCA1 by using the antibodies indicated at the left. The experiments were performed three times with similar results; representative blots are shown. **(c) **E2 inhibits DNA damage in MDA-MB-468 clones stably expressing ERα. Neomycin-resistant control clones (neo) were used as controls. ER-positive T47D neomycin-resistant control clones (T47D neo) were used for comparison. Cultures were treated with E2 or RA alone or in combination (E2/RA) as described in Materials and Methods. Vehicle-treated clones were used as negative controls (con). Relative DNA damage was quantified with the single-cell gel electrophoresis assay. Error bars indicate SEM. **(d) **Ectopic ER expression in ER-negative MDA-MB-468 cells is sufficient to mediate the effects of E2 on DNA repair activity. Neomycin-resistant control clones (neo) were used as controls. ER-positive T47D neomycin resistant control clones (T47D neo) were used for comparison. Cultures were treated with E2 or RA alone or in combination (E2/RA) as described in Materials and Methods. Vehicle-treated clones were used as negative controls (con). Relative DNA repair activity was quantified by the plasmid end-joining assay. Error bars indicate SEM. **(e) **E2 increases cell survival in MDA-MB-468 clones stably expressing ERα. Neomycin-resistant control clones (neo) were used as controls. ER-positive T47D neomycin-resistant control clones (T47D neo) were used for comparison. Cultures were treated with E2 or RA alone or in combination (E2/RA) as described in Materials and Methods. Vehicle-treated clones were used as negative controls (con). Apoptotic cells were identified by TdT-mediated dUTP nick end labelling assay. Error bars indicate SEM.

To determine whether ERα was sufficient to confer E2 mediated DNA damage repair and increased survival on ER-negative breast cancer cell lines, we stably transfected MDA-MB-468 cells with an ERα expression vector. Expression of ERα protein in these clones in comparison with MDA-MB-468 vector control cells and G418-resistant ER-positive T47D cells is shown in Fig. 5a. Ectopic ERα formed complexes with BRCA1 and CBP in E2-treated MDA-MB-468 clones to a similar degree to that observed in T47D cells (Fig. 5b). RARα failed to form complexes with BRCA1 in RA-treated cells. These clones were treated with E2 and RA alone or in combination before exposure to etoposide. As shown in Fig. 5c, ectopic ERα expression in MDA-MB-468 cells resulted in E2-mediated decreases in relative DNA damage levels of 25% (*p *< 0.002). This effect was also observed when E2 and RA were used in combination. ERα expression in MDA-MB-468 clones had no effect on RA-mediated DNA damage. G418-resistant MDA-MB-468 control clones did not exhibit E2-mediated decreases in relative DNA damage levels. The effects of E2 and RA in G418-resistant ER-positive T47D clones were similar to those observed in the parental cell line. Decreased DNA damage was correlated with increased DNA repair activity in E2-treated ERα-expressing MDA-MB-468 clones, as demonstrated by the end-joining assay (Fig. 5d). Results obtained with T47D and MDA-MB-468 G418-resistant control clones were similar to those observed in the parental cell lines. Increased resistance to etoposide and survival was also observed in the E2-treated MDA-MB-468 clones (Fig. 5e). Treatment with RA decreased cell survival to a degree similar to that observed in the MDA-MB-468 parental line. Results obtained with T47D and MDA-MB-468 control clones were similar to those observed for the parental cell lines. These results indicate that ectopic ERα expression was sufficient to produce the E2-mediated effects on relative DNA damage levels, DNA repair, and survival in etoposide-treated MDA-MB-468 clones.

## Discussion

One of the key findings of this study is the protective effects of E2 on ER-positive breast cancer cell lines after DNA damage. This effect was ER dependent because stable transfection of this expression vector into ER-negative breast cancer cell lines resulted in decreased DNA damage and increased survival when these cells were treated with E2 before etoposide. These results contrast with previous studies in which metabolites of E2 were shown to cause DNA damage by the formation of direct adducts or the generation of reactive oxygen species (reviewed in [32]). Increased oxidative DNA damage has been detected in target tissues after exposure to estrogen, and a low-activity form of catechol-*O*-methyltransferase has been associated with an increased risk of breast cancer. Glutathione-depleted MCF7 cells treated with E2 exhibited significant increases in formation of 8-oxo-2'-deoxyguanosine [33]. Treatment of MCF7 cells with E2 resulted in a decreased ability to metabolize peroxide and increased sensitivity to peroxide-induced DNA damage [34]. These effects were not observed in ER-negative breast cancer cell lines. Anti-estrogens have been shown to activate the detoxifying enzyme quinone reductase and protect against E2-mediated DNA damage [35]. Our present study does not rule out these DNA damage effects but suggests a new role for E2 in DNA damage repair and cell survival that is regulated by complex formation with coactivator proteins and BRCA1.

Double-strand DNA breaks have been shown to induce a number of growth factor signaling pathways [36]. However, we determined that the protective effects of E2 were not dependent on a number of upstream kinases and second messengers. It has been known for many years that ER is phosphorylated by MAPK [37]. Since then, ER has been shown to be a substrate for other kinases such as Cdk2 and Akt, which increase transcriptional activation of the receptor [38,39]. However, our data suggest that the actions of these kinases on ER transcriptional activation may not be required to protect breast cancer cell lines against DNA damage, and E2 did not induce the expression of double-strand break repair genes. It will be interesting to determine whether ER mutants lacking phosphorylation sites or transcriptional activation domains can inhibit the effects of E2 on double-strand break repair and breast cancer cell survival.

BRCA1 is phosphorylated by ATM kinase, which detects double-strand DNA breaks (reviewed in [40]). BRCA1 is phosphorylated at carboxyl-terminal serine residues and colocalizes with histone H2AX and Rad proteins at sites of double-strand break repair [41,42]. BRCA1-null cells are sensitive to double-strand breaks and are deficient in repairing this type of DNA damage [43,44]. BRCA1 represses E2-responsive gene expression and ER transcriptional activity, which may link the functions of BRCA1 to specific target tissues [45]. BRCA1 can bind directly to ER independently of E2 through the amino terminus of the tumor suppressor and the carboxyl domain of the receptor [46]. Amino-terminal truncations of BRCA1 blocked the ability of the tumor suppressor to inhibit ER activity in these studies. However, our results with a mutant BRCA1 protein showed that despite an intact amino terminus, the truncated tumor suppressor was not able to inhibit E2-mediated increases in double-strand break repair and cell survival. These data suggest a role for the BRCA1 carboxyl terminus in mediating the E2-dependent effects. We showed that this ligand-mediated protection was correlated with the formation of ER/coactivator complexes with BRCA1. However, treatment with RA did not recruit BRCA1 to RAR-CBP heterodimers, suggesting a receptor-specific effect. Our studies demonstrated that in the absence of the BRCT carboxyl domain, the mutant BRCA1 repressed the expression of multiple double-strand break repair proteins. Future studies will be important to examine the mechanisms by which these transcriptional complexes regulate DNA repair genes.

Our results show that the expression of a mutant BRCA1 construct inhibited cell cycle progression in human breast cancer cell lines, which correlated with decreased sensitivity to double-strand breaks. A previous study showed that loss of BRCA1 function in breast cancer resulted in cell cycle arrest through p53 and p21 [47]. In agreement with our results, several carboxyl-terminal truncated BRCA1 proteins conferred chemoresistance and decreased susceptibility to apoptosis [48]. However, a small carboxyl-terminal BRCA1 truncation caused defective transcriptional activation, cell cycle progression, and increased sensitivity to double-strand breaks in an ovarian cancer cell line [49]. These studies illustrate cell-specific differences in BRCA1 function and show that the carboxyl-terminal domain needs to be better defined if we are to understand its effects on these diverse cellular processes.

Our results demonstrated that treatment with E2 resulted in complex formation between ERα, CBP, and BRCA1 in ER-positive breast cancer cell lines. ERβ has been shown to inhibit the proliferation and E2-dependent stimulation of breast cancer cell lines [50,51]. It will be interesting to determine whether ERβ differentially affects the response to DNA damage in human breast cancer cells. Treatment with RA recruited CBP but not BRCA1 to RARα in both ER-positive and ER-negative cell lines. The carboxyl-terminal domain of CBP has been shown to interact *in vitro *and *in vivo *with BRCA1 [52]. BRCA1 interaction with CBP and p300 was shown to occur in a phosphorylation-independent manner through the CREB-binding domain of the coactivators and both the amino and carboxyl termini of the tumor suppressor [24]. The ability of BRCA1 to repress ER-responsive gene expression was correlated with its ability to downregulate the expression of p300 but not that of [53]. Increased expression of CBP or p300 rescued the inhibition of ER-responsive genes by BRCA1, perhaps by displacing BRCA1 from the nuclear receptor. Sequence comparisons between ER and RAR may reveal important differences between these receptors that functionally regulate their interactions with coactivators and BRCA1.

## Conclusion

E2 and RA had opposing effects on the survival of ER-positive breast cancer cell lines MCF7 and T47D after double-strand DNA break damage. Signaling pathways upstream of ER had no effect on the survival-promoting effect of E2. The cell survival effects of E2 and RA on the ER-positive human breast cancer cell lines were correlated with relative DNA damage levels in cultures treated with etoposide. The effects of E2 and RA on DNA damage were correlated with DNA repair activity in ER-positive human breast cancer cell lines. Treatment with E2 resulted in the formation of a complex between ERα, CBP, and BRCA1 in ER-positive breast cancer cell lines. Treatment with RA recruited CBP but not BRCA1 to RARα in both ER-positive cell lines and the ER-negative cell lines MDA-MB-231 and MDA-MB-468. Mutant BRCA1 expression reduced the expression of DNA damage repair proteins and was correlated with increased etoposide-mediated DNA damage in these lines but did not block nuclear hormone-dependent effects. Expression of the BRCA1 mutant resulted in decreased DNA repair activity in ER-positive and ER-negative breast cancer clones. Despite decreased DNA repair as the result of mutant BRCA1 expression, this construct produced increased survival in breast cancer cells with DNA double-strand breaks. The truncated BRCA1 failed to form complexes with ERα and CBP; this was correlated with its ability to exert E2-independent effects on DNA damage repair. The mutant BRCA1 construct, but not BRCA1 siRNA, inhibited cell cycle progression, which was correlated with increased resistance to etoposide. Ectopic ERα expression was sufficient to produce the E2-mediated effects on relative DNA damage levels, DNA repair, and survival in etoposide-treated MDA-MB-468 clones.

## Abbreviations

BRCA1 = breast cancer susceptibility gene 1; BRCT = BRCA1 carboxyl-terminal; BrdU = bromodeoxyuridine; CBP = CREB-binding protein; Cdk = cyclin-dependent kinase; E2 = 17β-estradiol; ER = estrogen receptor; RA = all-*trans *retinoic acid; RAR = retinoic acid receptor; siRNA = short interfering RNA; TUNEL = TdT-mediated dUTP nick end labelling.

## Competing interests

The authors declare that they have no competing interests.

## Authors' contributions

DLC conceived the study and directed the research. MKL contributed to the design of the study.
